# Attenuates of NAD^+^ impair BMSC osteogenesis and fracture repair through OXPHOS

**DOI:** 10.1186/s13287-022-02748-9

**Published:** 2022-02-22

**Authors:** Boer Li, Yu Shi, Mengyu Liu, Fanzi Wu, Xuchen Hu, Fanyuan Yu, Chenglin Wang, Ling Ye

**Affiliations:** 1 State Key Laboratory of Oral Diseases and National Clinical Research Center for Oral Diseases, West China Hospital of Stomatology, Sichuan University, Chengdu, China; 2 Department of Endodontics, West China Hospital of Stomatology, Sichuan University, Chengdu, China

**Keywords:** BMSC, Differentiation, NAD, OXPHOS, Energy metabolism

## Abstract

**Background:**

Controlling the adipo-osteogenic lineage commitment of bone marrow mesenchymal stem cell (BMSC) in favor of osteogenesis is considered a promising approach for bone regeneration and repair. Accumulating evidence indicates that oxidative phosphorylation (OXPHOS) is involved in regulating cell fate decisions. As an essential cofactor for OXPHOS, nicotinamide adenine dinucleotide (NAD) has been shown to correlate with the differentiation of stem cells. However, whether NAD manipulates BMSC lineage commitment through OXPHOS remains elusive. Therefore, it is critical to investigate the potential role of NAD on energy metabolism in mediating BMSC lineage commitment.

**Methods:**

In this study, the mitochondrial respiration and intracellular NAD^+^ level were firstly compared between osteogenic and adipogenic cells. For validating the role of NAD in mitochondrial OXPHOS, the inhibitor of NAD^+^ salvage pathway FK866 and activator P7C3 were used to manipulate the NAD^+^ level during osteogenesis. Furthermore, a murine femur fracture model was established to evaluate the effect of FK866 on bone fracture repair.

**Results:**

We elucidated that osteogenic committed BMSCs exhibited increased OXPHOS activity and a decreased glycolysis accompanied by an elevated intracellular NAD^+^ level. In contrast, adipogenic committed BMSCs showed little change in OXPHOS but an upregulated activity in glycolysis and a decline in intracellular NAD^+^ level in vitro. Moreover, attenuates of NAD^+^ via salvage pathway in BMSCs diminished osteogenic commitment due to mitochondria dysfunction and reduced activity of OXPHOS. The cells were rescued by supplementing with nicotinamide mononucleotide. In addition, treatment with NAD^+^ inhibitor FK866 impaired bone fracture healing in vivo.

**Conclusion:**

Our data reveals NAD^+^-mediated mitochondrial OXPHOS is indispensable for osteogenic commitment in BMSCs and bone repair, which might provide a potential therapeutic target for bone repair and regeneration.

**Supplementary Information:**

The online version contains supplementary material available at 10.1186/s13287-022-02748-9.

## Introduction

Bone marrow mesenchymal stem cells (BMSCs) are being exploited as seed cells for tissue regeneration and stem cell therapy because of their self-renewable capacity and the potential to differentiate into multiple types of cells. Osteogenesis and adipogenesis have been considered mutually exclusive in BMSCs differentiation. Maintaining the balance of BMSCs differentiation is critical for bone homeostasis but remains elusive.

A variety of factors contribute to the lineage commitment of BMSCs toward adipocyte or osteoblast formation, including extracellular environment, transcriptional factors, and cell metabolism [1]. Emerging evidence shows that the metabolic disorder in type 2 diabetes or obesity led to BMSCs differentiation dysfunction, suggesting metabolic changes and bioenergetic state were associated with BMSCs cell fate decision and cellular function [2, 3]. As is known, mitochondria are vital organelles that generate adenosine triphosphate (ATP) and a variety of metabolites. Oxidative phosphorylation (OXPHOS) is one of the most important metabolic activities of mitochondria. Studies found that OXPHOS coupled energy homeostasis could regulate cell fate decisions via the mTOR signaling pathway [4]. Besides, OXPHOS generated nicotinamide adenine dinucleotide (NAD^+^) and flavin adenine dinucleotide (FAD^+^), which was essential in the maintenance of glucose glycolysis, tricarboxylic acid cycle (TCA) cycle, and fatty acid β-oxidation [5, 6]. OXPHOS plays a critical role in energy homeostasis and is considered a potential target for multiple diseases [5, 7]. However, the role of OXPHOS in regulating BMSCs cell fate decision and differentiation was just begun to explore.

Current studies demonstrated that osteogenic differentiation of BMSCs was accompanied by mitochondrial OXPHOS increasing, but either no change or a decrease in glycolysis [8, 9]. Moreover, mitochondria transfer could enhance BMSCs osteogenesis and improve bone defect healing by upregulation of OXPHOS activity and ATP production [10]. On the contrary, a recent study reported that the osteogenic committed murine immortalized BMSCs maintained higher glycolytic activity but similar OXPHOS activity compared with adipogenic committed BMSCs [11]. In addition, aerobic glycolysis but not OXPHOS activity was increased during osteoblast differentiation and accounted for the majority of ATP production in mature osteoblasts [12]. These studies have confirmed that the cellular metabolism changes during BMSCs lineage commitment but with inconsistent results. Therefore, how to manipulate BMSCs differentiation through cellular metabolism needs further investigation.

As an essential component involved in bioenergetic pathways and posttranslational modifications, nicotinamide adenine dinucleotide (NAD) has emerged as a potential regulator of cellular metabolism and cell function [13, 14], which participates in various energy metabolism pathways, including glycolysis, β-oxidation, and OXPHOS. NAD exists in oxidized and reduced forms, abbreviated as NAD^+^ and NADH, respectively. Of note, an optimal intracellular NAD^+^ level and NAD^+^/NADH ratio are essential for mitochondrial function [15]. The NAD^+^ is also an obligatory substrate for the reaction of glycolysis and OXPHOS. Studies have shown that NAD^+^/NADH ratio is involved in metabolic change. The stem cells maintain a relatively low NAD^+^/NADH and mitochondrial potential, reflecting their glycolysis-driven metabolic profile [16]. The deficiency of NAD^+^ suppresses activities of NAD (H)-dependent enzymes in glycolysis and OXPHOS, which leads to the stagnation of glycolytic flux and ATP production [17]. Furthermore, a historical study has reported that aging-related osteoporosis was associated with a decline in NAD^+^ level [18]. The restoration of NAD^+^ decelerated the bone loss in the aging process with enhanced osteogenesis and suppressed adipogenesis via Sirt1-dependent pathway [19, 20]. Additionally, supplements of primary NAD^+^ precursors, nicotinamide (NAM) or nicotinamide mononucleotide (NMN), could regulate BMSCs cell function via boosting intracellular NAD^+^ level [21, 22]. However, they were unable to address the role of NAD^+^-dependent metabolic regulation on BMSCs lineage commitment. Besides, the changes of NAD^+^/NADH during lineage commitment remain unclear. Whether NAD^+^ and NAD^+^/NADH redox state could influence the lineage commitment of BMSCs by altering energy metabolism needs further investigation.

In the present study, we have investigated the metabolic feature during adipogenesis and osteogenesis of BMSCs. We report that osteogenic committed BMSCs possessed an increased OXPHOS activity and a decreased glycolysis along with an elevated NAD^+^ level. In comparison, adipogenic committed cells exhibited an upregulated activity in glycolysis but with a decline in intracellular NAD^+^ level in vitro. Importantly, suppression of NAD^+^ via salvage pathway impaired mitochondrial fusion, leading to mitochondria dysfunction and reduced activity of OXPHOS, which subsequently blocked osteogenesis. Moreover, inhibition of NAD^+^ diminished bone fracture healing in vivo. Taken together, NAD^+^-mediated OXPHOS is indispensable for osteoblastogenesis and bone regeneration.

## Methods

### Cell culture

Human bone marrow-derived mesenchymal stem cells (hBMSCs) were purchased from Cyagen Bioscience company (China). Murine bone marrow stromal cell line ST2 was purchased from Jennio Biotech company (China). Cells were expanded in aMEM medium (Gibco) with 10% fetal bovine serum (Gibco) and 1% penicillin–streptomycin (Hyclone). The medium was changed every other day. For all experiments, we used hBMSCs from passage 4–7 and ST2 cell line from passage 4–8 as recommended by the manufacturer. The hBMSCs were seeded at 2 × 10^4^ cells/cm^2^ and the ST2 cells were seeded at 1 × 10^4^ cells/cm^2^ for osteogenic and adipogenic induction.

Osteogenesis of hBMSCs or ST2 cells was induced with 50 μg/ml ascorbic acid, 10 mM β-glycerophosphate, and 10 nM dexamethasone (all from Sigma, USA). To confirm osteogenesis and mineralization, we performed alkaline phosphatase (ALP) stain (Beyotime Biotechnology, China) and 2% alizarin red stain (Beyotime Biotechnology, China) on Day 7 or Day 14, respectively. The density of ALP stain and alizarin red stain was quantified by Image Pro Plus 6.0. To induce adipogenesis, we cultured the cells with the addition of 0.5 mM 3-Isobutyl-1-methylxanthine (IBMX), 1 μg/ml insulin, and 100 μM dexamethasone (all from Sigma, USA) for 3 days, then changed induction medium with 1 μg/ml insulin. Oli red (Sigma, USA) stain was performed on Day 7 to confirm the formation of lipid droplets.

### Glucose consumption assay

Cells were cultured in custom-made media (Sigma, USA) containing 5.5 mM glucose, 2 mM glutamine, 0.1 mM pyruvate and 10% FBS for 24 h. The medium was collected and used for glucose or lactate measurement with Glucose (HK) Assay Kit (

Sigma-Aldrich, USA) or Lactate Assay Kit (Eton biosciences, USA), respectively. The cell number was counted for normalization.

### Seahorse metabolic profile assay

hBMSCs or ST2 cells were cultured and induced with osteogenic media or adipogenic media. After 7 days of induction, the cells were trypsinized and seeded at 4 × 10^4^ cells/well in XF24 plates coated with poly-D-lysine for 2 h. On the same day, the cells were changed with XF base medium supplemented with 5.5 mM glucose, 2 mM glutamine, and 0.1 mM pyruvate and further incubated in a CO_2_-free incubator for 1 h. For Seahorse XF cell mito-stress test, oligomycin, trifluoromethoxy carbonylcyanide phenylhydrazone (FCCP), antimycin A, and rotenone (Seahorse XF Cell Mito Stress Test Kit, Seahorse Bioscience, 103015–100) were prepared in XF base medium and used at the final concentrations of 1.5 µM for oligomycin and FCCP, and 1 µM for rotenone and antimycin A. For Seahorse XF glycolytic rate assay, 1 µM for rotenone and antimycin A and 50 mM 2-Deoxy-D-glucose (2DG) (Seahorse XF Glycolytic Rate Assay Kit, Seahorse Bioscience, 103344–100) were prepared. The cell number was used to normalize oxygen consumption rate (OCR) and extracellular acidification rate (ECAR)**.** The following OXPHOS and glycolytic indexes were calculated: basal respiration (OCR_pre-Olig_ − OCR_post-AntA_), ATP-linked respiration (OCR_pre-Olig_ − OCR_post-Olig_), maximal respiration (OCR_post-FCCP_ − OCR_post-AntA_), spare respiratory (OCR_postFCCP_ − OCR_pre-Olig_), proton leak (OCR_post-Olig_ − OCR_post-AntA_), and basic glycolysis (ECAR_pre-Olig_).

### JC-1 staining for mitochondria membrane potential

JC-1, a cationic dye, was used to assess the mitochondrial membrane potential (ΔΨ_M_) changes. In brief, hBMSCs or ST2 cells were cultured in medium containing 10 μg/ml JC-1 (Beyotime Biotechnology, China) at 37 °C for 20 min. As a pharmacological control, cells were pretreated with 10 μM carbonyl cyanide 3-chlorophenylhydrazone (CCCP) (Beyotime Biotechnology, China) for 20 min and then stained with JC-1. Following washes, the cells were cultured in growth medium and examined by fluorescence microscopy (Leica, German), confocal microscopy (Olympus FV3000, Japan), or flow cytometry (Thermo Attune Nxt, USA). The captured images from three independent experiments were processed using Image J software to measure the red and green fluorescence intensity. When the ΔΨ_M_ is high, JC-1 aggregates to form polymers J-aggregates in the mitochondria and produce red fluorescence. When the ΔΨ_M_ is low, JC-1 cannot aggregate in the mitochondrial matrix and remains the form of monomer to produce green fluorescence. The mitochondrial membrane potential (ΔΨ_M_) was evaluated by calculating the ratios of red/green fluorescence intensity. The FACS images were processed via FlowJo 10 (FlowJo LLC, USA).

### Mito-tracker staining

hBMSCs or ST2 cells were incubated in 250 nM MitoTracker Red FM (Thermo, USA) with 5 μg/ml Hoechst 33342 (Thermo, USA) at 37 °C for 30 min. Cells were cultured in growth medium following washes, and the mitochondrial morphology was examined by confocal microscopy (Olympus FV3000, Japan).

### Citrate synthase activity assay

Citrate synthase (CS) activity is a frequently used biomarker of mitochondrial content [23]. The mitochondrial protein was isolated with the lysis buffer. The CS activity was measured with the mitochondrial suspension by citrate synthase activity kit (Solarbio Science & Technology Co., Ltd., China). The protein content was measured for normalization.

### Quantitative real-time PCR

Total RNA of the cells was extracted using the RNeasy mini kit (Qiagen, USA). Complementary DNA was synthesized using the HiScript II cDNA synthesis kit (Vazyme Biotech Co., Ltd, China). ChamQ Universal SYBR qPCR Master Mix (Vazyme Biotech Co., Ltd, China) was used for qPCR reaction. The nucleotide sequence of primers is listed in Table 1. Beta-actin (*β-actin)* was used as an internal control. The relative expression level of mRNA was calculated by the 2-(ΔΔCT) method and presented as fold changes relative to *β-actin* [24].

**Table 1:** Nucleotide sequence of PCR primers (Homo sapiens)

Gene	Primer F/R	Sequence 5′–3′
*RUNX2*	F	GACTGTGGTTACCGTCATGGC
*RUNX2*	R	ACTTGGTTTTTCATAACAGCGGA
*ALPL*	F	GACCTCCTCGGAAGACACTC
*ALPL*	R	TGAAGGGCTTCTTGTCTGTG
*SP7*	F	TCTCCATCTGCCTGACTCCT
*SP7*	R	AGCGTATGGCTTCTTTGTGC
*COL1α1*	F	TCTAGACATGTTCAGCTTTGTGGAC
*COL1α1*	R	TCTGTACGCAGGTGATTGGTG
*BGLAP*	F	TCACACTCCTCGCCCTATTG
*BGLAP*	R	GGGTCTCTTCACTACCTCGC
*SPP1*	F	AGCTTTACAACAAATACCCAGATGC
*SPP1*	R	GGACTTACTTGGAAGGGTCTGTG
*CEBPα*	F	CCAGAAAGCTAGGTCGTGGG
*CEBPα*	R	TCCTAGGCAATGCTGAAGGC
*PPARγ*	F	GGGATCAGCTCCGTGGATCT
*PPARγ*	R	TGCACTTTGGTACTCTTGAAGTT
*NAMPT*	F	CTTCGGTTCTGGTGGAGGTT
*NAMPT*	R	ATCGGCCCTTTTTGGACCTT
*β-actin*	F	TCACTATTGGCAACGAGCG
*β-actin*	R	AGGTCTTTACGGATGTCAACG

### Western blotting

The total protein for the cells was extracted using M-PER™ Mammalian Protein Extraction Reagent (Thermo Scientific, IL, USA) with Halt™ Protease and Phosphatase Inhibitor Cocktail (Thermo Scientific, IL, USA). 20 µg protein for each sample was loaded on a 10% or 15% polyacrylamide gel and then transferred to the 0.22 μm PVDF membrane (Millipore, USA). After being blocked for 1 h with 5% nonfat milk in triethanolamine buffered saline and 0.5%Tween-20 (TBST), the PVDF membranes were incubated overnight at 4 °C with primary antibodies. The primary antibodies included anti-MFN1 (1:1000, #13798-1-AP, Proteintech, China), anti-MFN2 (1:1000, #12186-1-AP, Proteintech, China), anti-FIS1 (1:1000, #10956-1-AP, Proteintech, China), anti-NAMPT (1:250, #ab45890, Abcam, USA), anti-β-ACTIN (1:5000, #HRP-60008, Proteintech, China). This was followed by a 1-h incubation with appropriate horseradish peroxidase (HRP)-conjugated IgG antibodies (Abcam, USA). Subsequently, PVDF membranes were washed three times with TBST, and proteins were visualized with Luminata Forte Western HRP substrate (Millipore Corp., MA, USA). Quantification of the integrated density was performed with three independent experiments by Image J software.

### NAD^+^/NADH assay kit

hBMSCs or ST2 cells were collected and lysed with NAD/NADH extraction buffer. Then the extracted NAD^+^/NADH supernatant was used for NAD^+^/NADH measurement with NAD^+^/NADH assay kit (Beyotime Biotechnology, China). The cell number was counted for normalization.

### Mitochondria transmission electron microscope analysis

Cells were fixed with 2.5% glutaraldehyde at 4 °C for 10 min and subsequently centrifuged at 2000 rpm for 10 min. The cell pellets were post-fixed with 3% glutaraldehyde. After post-fixation, the cell pellets were rinsed twice in H_2_O at 4 °C, dehydrated through a graded series of alcohol at 4–20 °C, infiltrated with graded mixtures of propylene oxide (substituted by acetone in 2 samples) and Epon at 20 °C, and embedded in 100% Epon at 30 °C. The ultra-thin sections were cut in three depths separated by 150 nm. The sections were contrasted with uranyl acetate and lead citrate and examined and photographed in a pre-calibrated Philips EM 208 electron microscope and a Megaview III FW camera (both FEI Company, Eindhoven, the Netherlands).

### Murine femur fracture surgery

8-Week-old mice were anesthetized by isoflurane inhalation anesthesia. The right femur was then shaved and scrubbed with betadine. All instruments and pin implants were sterilized before use. A 1 cm surgical incision was made over the anterolateral distal femur to expose the mid-point femur. A mid-shaft transverse fracture was made using a sharp scalpel. A 24-gauge stainless-steel pin was passed into the intramedullary canal to stabilize the fracture with the keen flexed. Radiographs were taken immediately after surgery and before sacrifice to confirm pin placement and fracture pattern. Animals were given buprenorphine (0.05 mg/kg) to alleviate any surgical pain. Sutures were checked daily for 3 days and removed on day 7.

Mice were randomly allocated into either vehicle (DMSO) or FK866 group and received intraperitoneal (IP) injections of FK866 (10 mg/kg, Sigma-Aldrich, USA) or vehicle in pyrogen-free saline every 12 h from the first postoperative day. On day 21, mice were euthanized and prepared for analysis.

### μCT analysis

Bone tissues were dissected and fixed in 4% paraformaldehyde for 48 h at 4 °C and sorted in 70% ethanol for further experiments. The microCT imaging system (μCT50, SCANCO Medical, 10 μm voxel size, 70kVp, 114μA) was applied to evaluate fracture callus. Total volume (TV), bone volume (BV), and volumetric bone mineral density (BMD) were analyzed. Femurs were used for histology analysis after scanning.

### Histomorphometric analyses

Bone specimens were decalcified with 14%EDTA for 2 weeks. The femurs were embedded in paraffin, sectioned at 5 μm, and stained with hematoxylin and eosin (H&E, Solarbio Science & Technology Co., Ltd., China), Goldner trichrome stain (Solarbio Science & Technology Co., Ltd., China) following manufacture’s protocol.

For Immunohistochemistry (IHC) stain, the sections were performed proteolytic-induced antigen retrieval and subsequently stained with HRP-DAB Cell & Tissue Staining Kit (R&D, USA). Appropriate primary antibodies, anti-NAMPT (1:200, #R27427, ZENBIO, China), anti-Osterix (1:100, #67138, Abcam, USA), and anti-Aggrecan (1:100, #ab1031, Millipore, USA) were used. Images were obtained using a Nikon Eclipse 300 microscope (Compix Inc, Sewickley, PA).

### Statistics

One-way or two-way ANOVA with post-hoc Tukey correction was carried out for comparisons of multiple groups. Student’s t test was performed to determine the statistical significance for two groups. A *p* value < 0.05 was considered to be significant. Numerical data and histograms were expressed as mean ± SD (standard deviations). Results were presented in the presence of at least three independent biological experiments, and for each experiment, at least three technical repeats were carried out.

## Results

### Distinct metabolic profile and mitochondrial function in osteogenic and adipogenic committed BMSC

To study the potential metabolic distinction of BMSCs during osteogenesis and adipogenesis, we cultured human BMSCs and bone marrow stromal cell line ST2 to induce differentiation. Flow cytometry analysis was used to identify the undifferentiated hBMSCs and showed that the majority of the cells were CD31^−^CD45^−^CD73^+^ (Additional file 1: Fig. S1A). Alizarin red staining, and oil red staining were performed to confirm the osteogenic and adipogenic differentiation, respectively (Additional file 1: Fig. S1B, C). In addition, molecular analysis showed that the known differentiation markers were upregulated during induction as expected (Additional file 1: Fig. S1D, E).

**Fig. 1:**
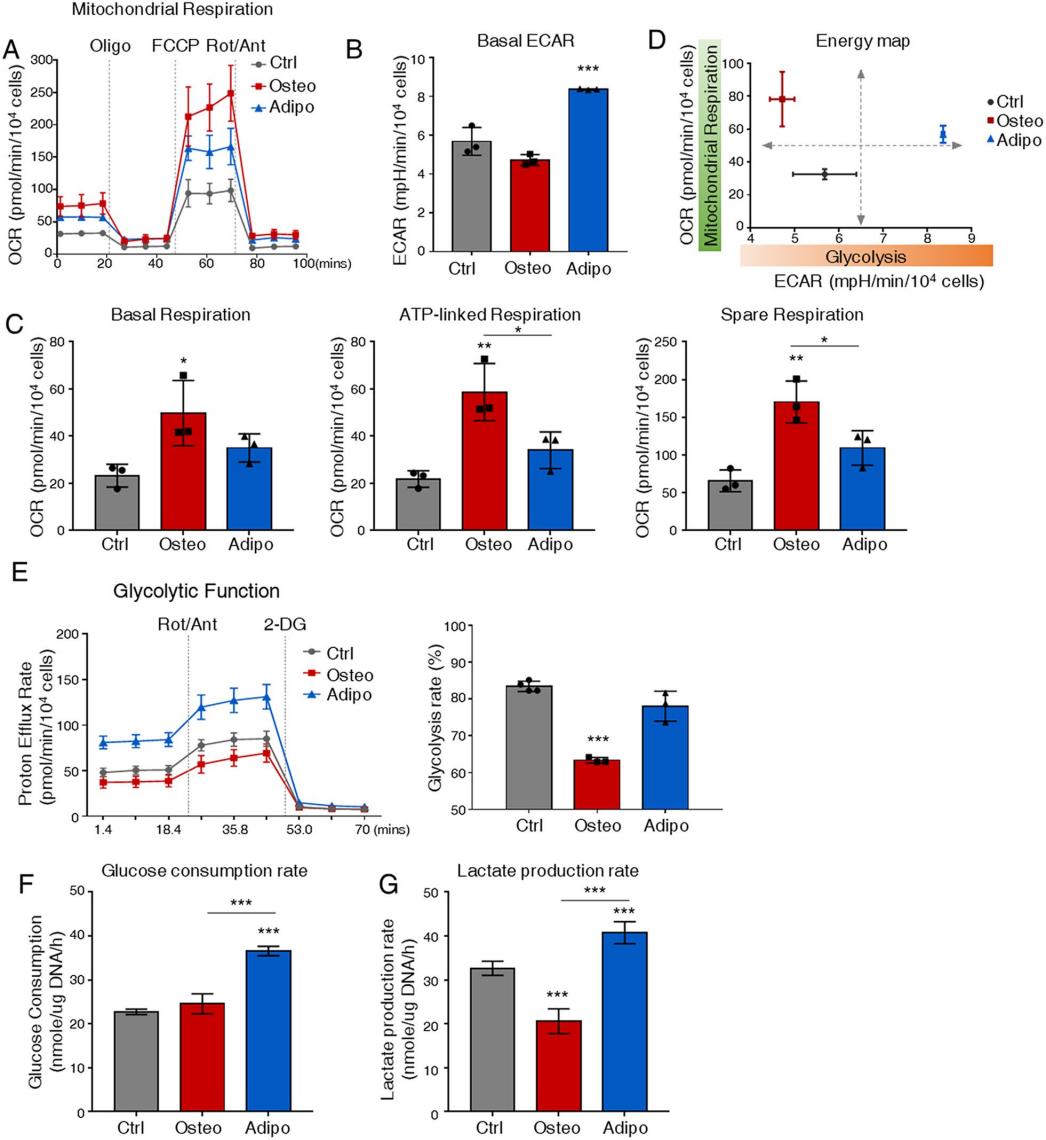
Distinct metabolic profiles in osteogenic and adipogenic committed BMSCs. **A** Seahorse mito-stress test for oxygen consumption rate (OCR) in BMSCs on Day 7. **B** Seahorse mito-stress test for extracellular acidification rate (ECAR) in BMSCs on Day 7. **C** Quantification of OCR parameters in BMSCs. **D** Energy map of BMSCs. Mean basal OCR in dependence of mean basal ECAR is shown. **E** Seahorse glycolytic test and quantification for ECAR in ST2 cells on Day 7. **F** Glucose consumption rate in BMSCs on Day 7. **G** Lactate production rate in BMSCs on Day 7. Ctrl: control; Osteo: osteogenesis; Adipo: adipogenesis; Oligo: oligomycin; FCCP: Trifluoromethoxy carbonylcyanide phenylhydrazone; Rot: rotenone; Ant: antimycin A. *N* = 3. Statistical significance was calculated with one-way ANOVA. *p < 0.05; **p < 0.01. Data are represented as mean ± SD

Firstly, we performed the cell mitochondrial stress test with seahorse analyzer. BMSCs or ST2 cells were induced with osteogenic or adipogenic media for 7 days and then used for Seahorse assays. Compared to undifferentiated cells, osteogenic committed hBMSCs exhibited a notable increase in the basal oxygen consumption rate (OCR) but little change in basal extracellular acidification rate (ECAR) (Fig. 1A, B). Mitochondrial stress tests showed that basal respiration, OCR related to ATP production, and spare capacity were all significantly increased in osteogenic BMSCs over undifferentiated cells or adipogenic BMSCs (Fig. 1C), suggesting that under conditions of increased energetic demand, osteogenic committed cells had a higher capacity to increase ATP synthesis through mitochondrial OXPHOS. On the other hand, the adipogenic committed BMSCs showed an increase in basal ECAR level without any significant changes in OCR measurement (Fig. 1A, B). The energy map based on the OCR and ECAR demonstrated that undifferentiated BMSCs showed lower energy levels while differentiated cells had a higher OCR or ECAR level, which may corroborate the functional demand. Besides, the osteogenic committed BMSCs tend to be more aerobic, and adipogenic committed BMSCs were more energetic (Fig. 1D). Consistently, the osteogenic ST2 cells showed an increase in OCR, whereas the adipogenic ST2 cells exhibited a comparable OCR level and elevated basal ECAR level compared with undifferentiated ST2 cells (Additional file 1: Fig. S2A). Furthermore, the seahorse glycolytic rate assay of the ST2 cell line showed that the glycolytic rate was dramatically reduced by 24% during osteogenesis (Fig. 1E), indicating that the glycolytic flux receded during osteogenesis.

To further investigate the changes in glucose flux, we detected the glucose consumption rate (GCR) and lactate production rate (LPR) in committed cells. The glucose consumption rate increased in adipogenic committed BMSCs and ST2 cells, whereas barely changed in osteogenic committed cells (Fig. 1F, Additional file 1: Fig. S2B). The lactate production rate was decreased in osteogenic cells but increased in adipogenic cells, consistent with the ECAR (Fig. 1G, Additional file 1: Fig. S2C). Altogether, our data illustrated distinct metabolic signatures of osteogenic and adipogenic committed hBMSCs. Comparative analysis of metabolic signatures of the hBMSCs in osteogenic or adipogenic induction revealed that osteogenic committed cells had higher activity of mitochondrial respiration and adipogenic committed cells had elevated glycolysis rate as evident by OCR, ECAR, and the energy map.

**Fig. 2:**
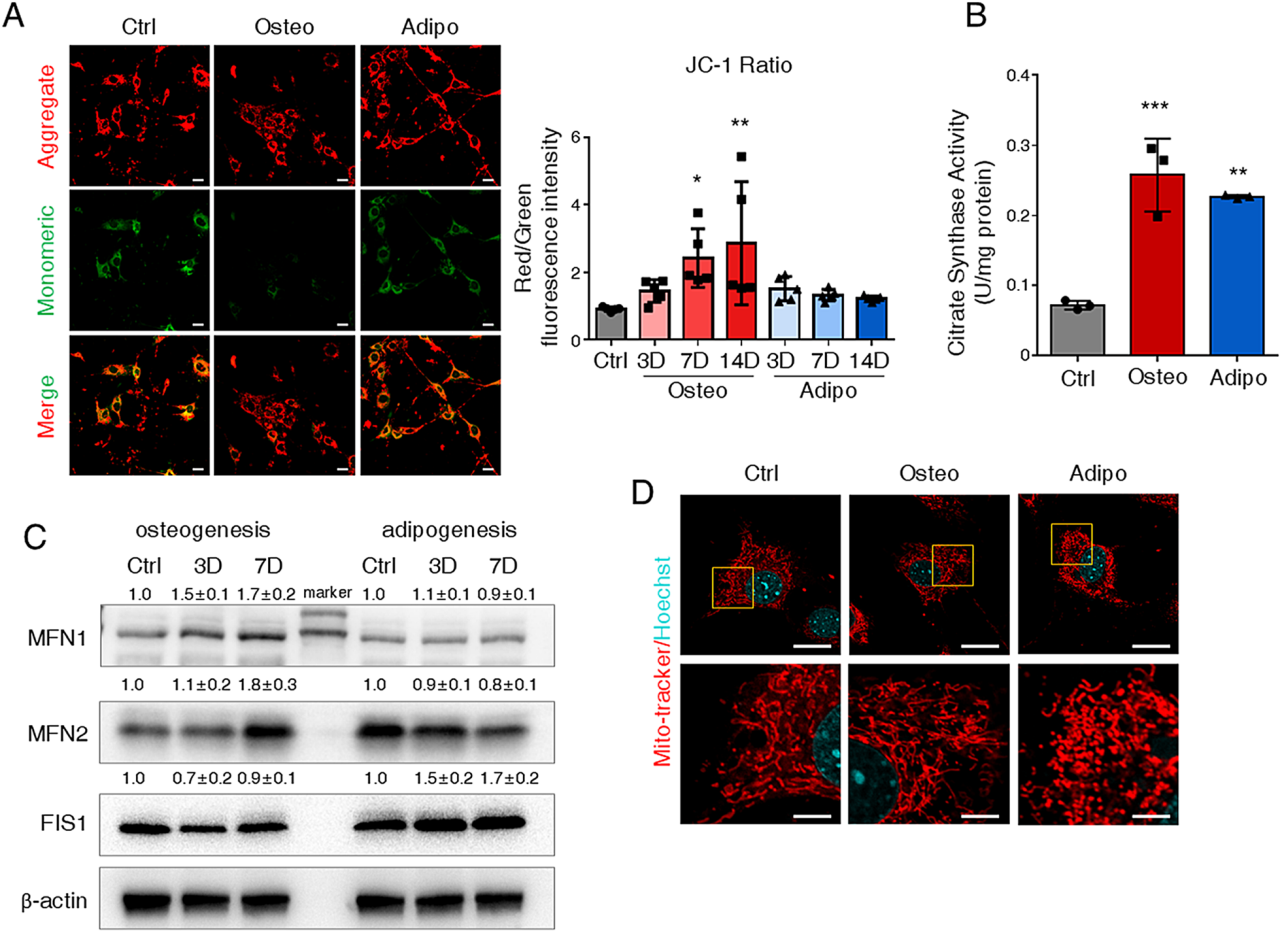
Mitochondrial function is enhanced in osteogenic and adipogenic committed cells. **A** Representative image of JC-1 staining in ST2 cells at day 7. Right: quantification of red/green fluorescence intensity ratio in ST2 cells. Scale bar = 50 μm. *N* = 4. **B** Citrate synthase activity in ST2 cells at day 7. *N* = 3. **C** Representative image of western blot analysis with the ST2 cells. Quantification of the western blot from three independent experiments was shown upon the bands. *N* = 3. **D** Mito-tracker staining for ST2 cells at day 7. Top panel scale bar = 20 μm, bottom panel scale bar = 5 μm. *N* = 4. 3D: day 3; 7D: day 7. 14D: day 14. Statistical significance was calculated with two-way ANOVA for (**A**) and one-way ANOVA for (**B**). *p < 0.05; **p < 0.01. Data are represented as mean ± SD

To gain further insights into the changes of mitochondrial respiration during differentiation, we next evaluated mitochondrial function by JC1 staining, mitochondrial mass, and morphology. JC-1-staining and quantification confirmed that mitochondrial membrane potential (ΔΨm) was gradually elevated during osteogenesis but did not change during adipogenesis in ST2 cells indicated by the ratio of red/green fluorescence intensity (Fig. 2A). Meanwhile, the mitochondrial mass was approximately a 3.5-folds increase in osteogenic cells and a threefold increase in adipogenic cells assessed by the citrate synthase activity [23] (Fig. 2B). As mitochondrial function was tightly correlated with mitochondrial morphology [25], we detected the expression of mitochondrial fusion and fission protein. Mitofusin-1 (MFN1), responsible for mitochondria fusion, was upregulated during osteogenesis, whereas no difference during adipogenesis. In contrast, the expression of fission-related protein Fission 1 (FIS1) was increased in adipogenic cells but not changed in osteogenic committed cells (Fig. 2C). Confocal images with mito-tracker-staining of mitochondria also demonstrated that alterations of mitochondrial morphology occurred during differentiation, which maintained punctated morphology in response to adipogenesis, whereas became elongated during osteogenesis (Fig. 2D). Collectively, these data revealed osteogenic cells exhibited elevated OXPHOS activity along with enhanced mitochondrial function, while adipogenic cells had an increased OXPHOS and glycolysis.

### NAD^+^ is indispensable for BMSC osteogenic commitment as a positive regulator

NAD^+^ is not only a cofactor involved in several redox reactions but also rewires cell metabolism and maintains mitochondrial fitness [26–28]. Besides, The NAD^+^ level and biosynthesis are coupled with energy metabolic changes [14].

**Fig. 3:**
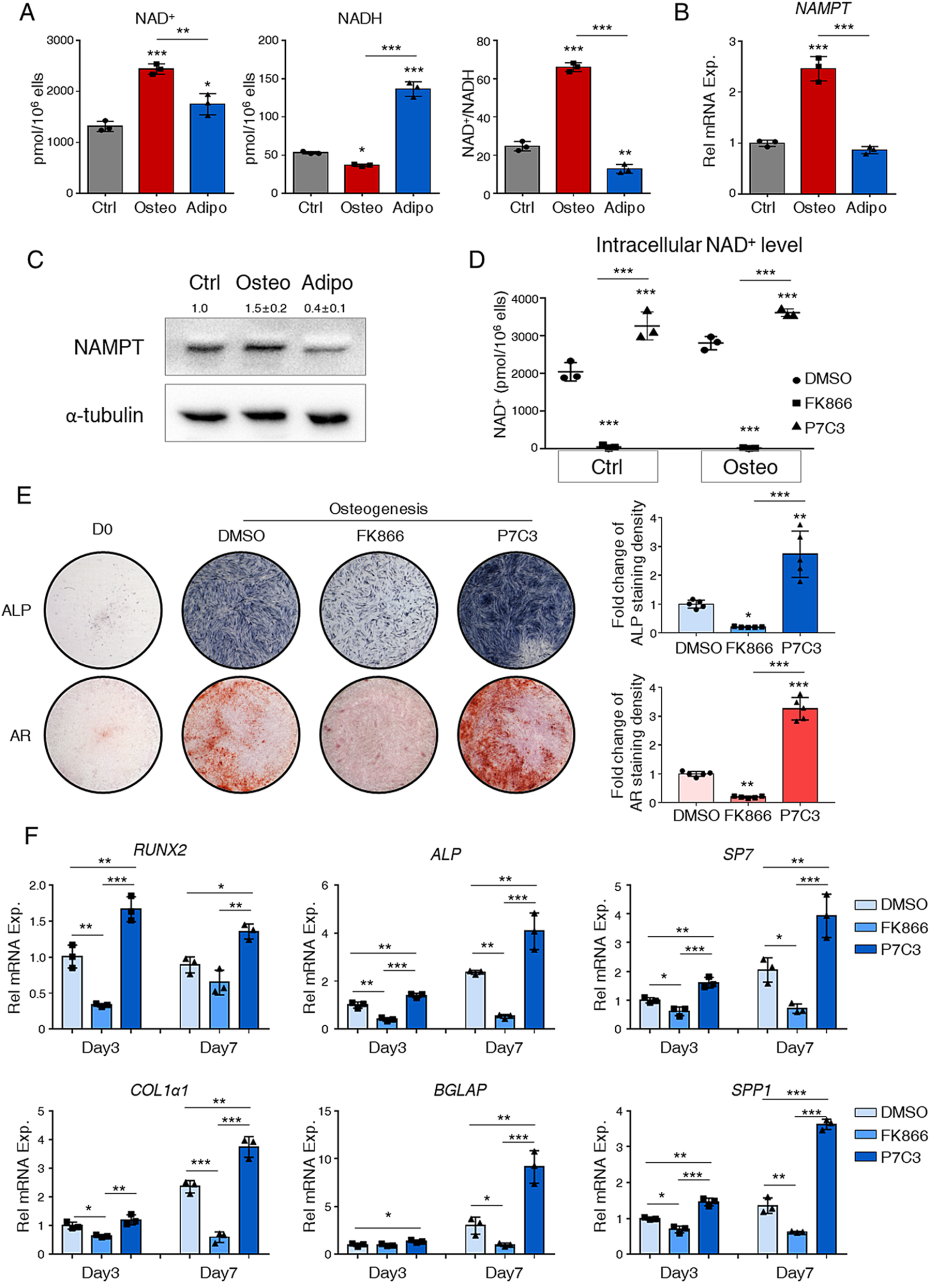
NAD^+^ is indispensable for BMSC osteogenic commitment. **A** Intracellular level of NAD^+^, NADH, and the ratio of NAD^+^/NADH in hBMSCs at day 7. **B** Relative expression of *NAMPT* in hBMSCs at day 7. **C** Representative image of western blot analysis with the hBMSCs at day 7. Quantification of the western blot from three independent experiments was shown upon the bands. *N* = 3. **D** Intracellular level of NAD^+^ of hBMSCs with vehicle (DMSO), FK866, or P7C3 treatment for 7 days. **E** Representative image of ALP staining (top) and Alizarin Red staining (bottom) for hBMSCs with DMSO, FK866, or P7C3 treatment. The staining of hBMSCs at D0 was presented as control. Right panels were quantification of the ALP or alizarin red staining from four independent experiments. *N* = 4. **F** Relative expression of osteogenic-related genes *RUNX2*, *ALP*, *SP7, COL1α1*, *BGLAP* and *SPP1* in hBMSCs with P7C3 or FK9866 treatment at day 3 and day 7. DMSO group at day 3 was used as control. *N* = 3. Statistical significance was calculated with one-way ANOVA. *p < 0.05; **p < 0.01; ***p < 0.001. Data are represented as mean ± SD

We observed dramatic increases in intracellular NAD^+^ level and NAD^+^/NADH in osteogenic committed hBMSCs (Fig. 3A). In contrast, adipogenic committed cells had higher NADH levels and decreased NAD^+^/NADH (Fig. 3A). Next, we detected the expression of related regulators in NAD^+^ biosynthesis and salvage pathway since it predominantly governed the intracellular NAD^+^ pool [26, 29, 30]. Of note, we found that nicotinamide phosphoribosyltransferase (NAMPT), which catalyzes the first reversible step in NAD^+^ biosynthesis and NAM salvage, was remarkably increased in osteogenic hBMSCs but reduced in adipogenic cells (Fig. 3B, C).

To further explore the role of NAMPT and NAD^+^ in hBMSCs osteogenesis, we administrated NAMPT activator P7C3 and inhibitor FK866 during differentiation. As expected, P7C3 elevated intracellular NAD^+^ level while FK866 dramatically inhibited NAD^+^ level (Fig. 3D). Functionally, P7C3 promoted hBMSCs osteogenesis and mineralized nodules formation, while FK866 suppressed osteogenesis of hBMSCs at day 7 and abolished the formation of mineralized nodules as stained by alizarin red at day 14 (Fig. 3E). Molecular analyses demonstrated that osteogenesis-related markers were downregulated by FK866 but upregulated by P7C3 after 3 days’ or 7 days’ osteogenic induction (Fig. 3F). Besides, FK866 could prevent spontaneous differentiation in hBMSC without osteogenic induction (Additional file 1: Fig. S3). These data indicated that intracellular NAD^+^ was essential for hBMSCs osteogenesis.

### Attenuates of NAD^+^ with FK866 impaired bone fracture repair

**Fig. 4:**
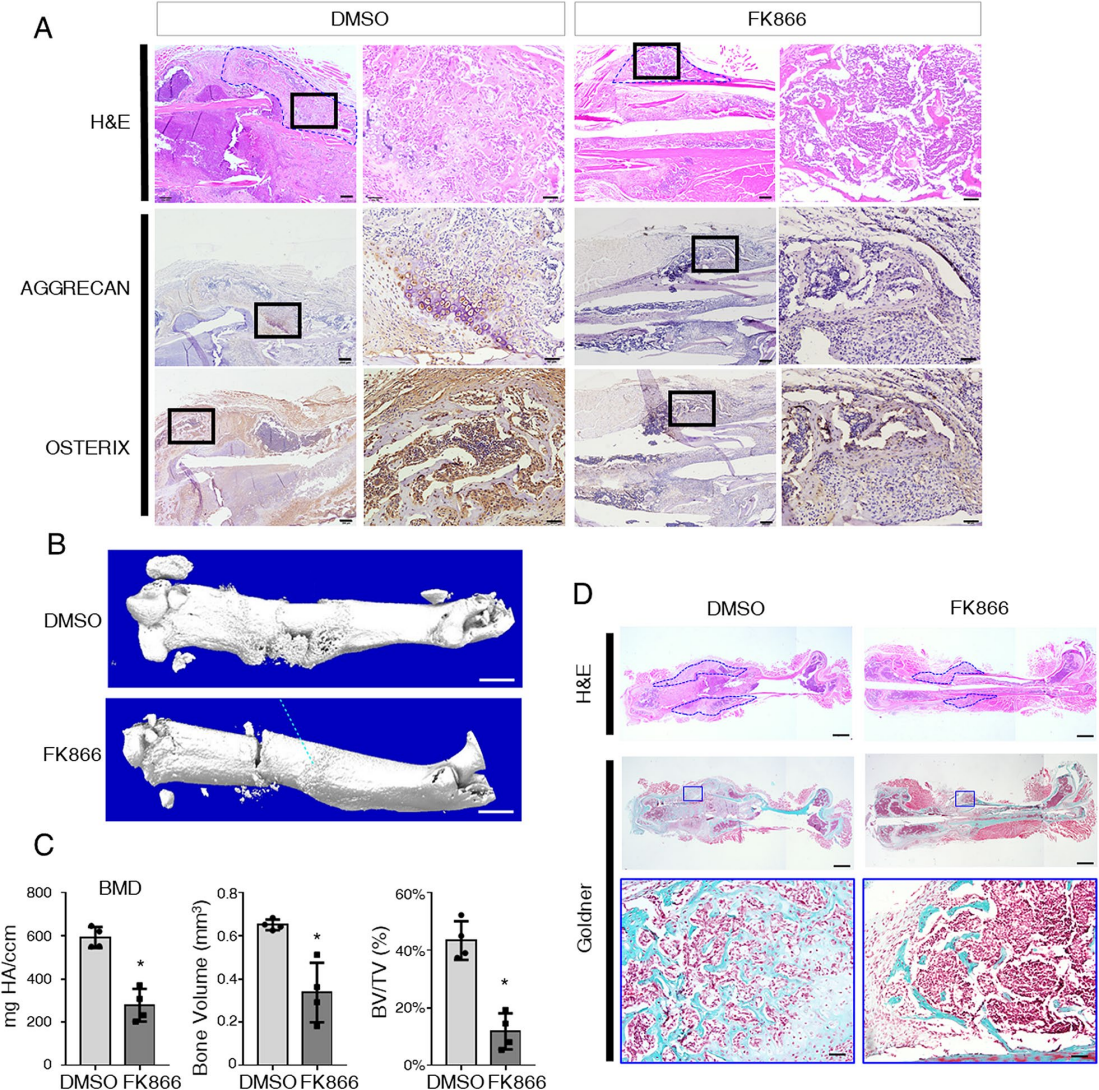
FK866 inhibits bone fracture repair in vivo. **A** Representative H&E stain and IHC stain of Aggrecan and Osterix at 3 weeks post-fracture. Scale bar in low magnification: 200 μm, scale bar in high magnification: 50 μm. The blue dash line delineated the bone callus. **B** μCT images at 3 weeks post-fracture. Scale bar = 1 mm. **C** Bone parameters from fracture callus at 3 weeks post-fracture by μCT. *N* = 4. BMD: bone mineral density. BV: bone volume. TV: total volume. **D** Representative H&E stain and goldner trichrome stain at 3 weeks post-fracture. The blue dash line delineated the bone callus. The blue-coded boxed areas in the low magnification images (upper) were shown at high magnification in the corresponding boxes (lower). Top scale bar = 1 mm, bottom scale bar = 200 μm. *N* = 4. Statistical significance was calculated with Student’s *t* test. *p < 0.05; **p < 0.01. Data are represented as mean ± SD

Since osteogenesis of BMSCs make an essential contribution to fracture healing, we next performed the mice femoral fracture model and sought to explore the role of NAMPT in osteogenesis in vivo. To investigate whether NAMPT was expressed during the bone fracture healing, we observed the bone callus at day 14, day 28, and day 42 post-fracture. At day 14, the expression of NAMPT in callus was barely detectable by immunostaining. Subsequently, NAMPT expression was detected 28 days after fracture, and it was broadly expressed in bony callus 42 days post-fracture (Additional file 1: Fig. S4). This data suggested a regulatory effect of NAMPT in bone regeneration in vivo. To further explore the role of NAD^+^ in bone formation, we used FK866 to specifically inhibit global NAMPT activity by intraperitoneal injection. Specifically, immunostaining confirmed FK866 severely diminished the expression of chondrogenesis marker Aggrecan and osteogenesis marker Osterix in callus (Fig. 4A). Micro-CT analyses and H&E staining showed a significant decrease in bone mineral density and smaller bone volume with the injection of FK866 in callus (Fig. 4B, C). Accordingly, FK866 impaired calcified tissue formation (shown in blue) in callus stained by Goldner-Trichrome (Fig. 4D). Thus, our data to date demonstrated inhibition of NAD^+^ with FK866 suppressed the bone fracture healing, indicating that intracellular NAD^+^ played as a positive regulator on BMSCs osteogenesis and bone repair.

### FK866 repressed osteogenesis through mitochondrial OXPHOS

**Fig. 5:**
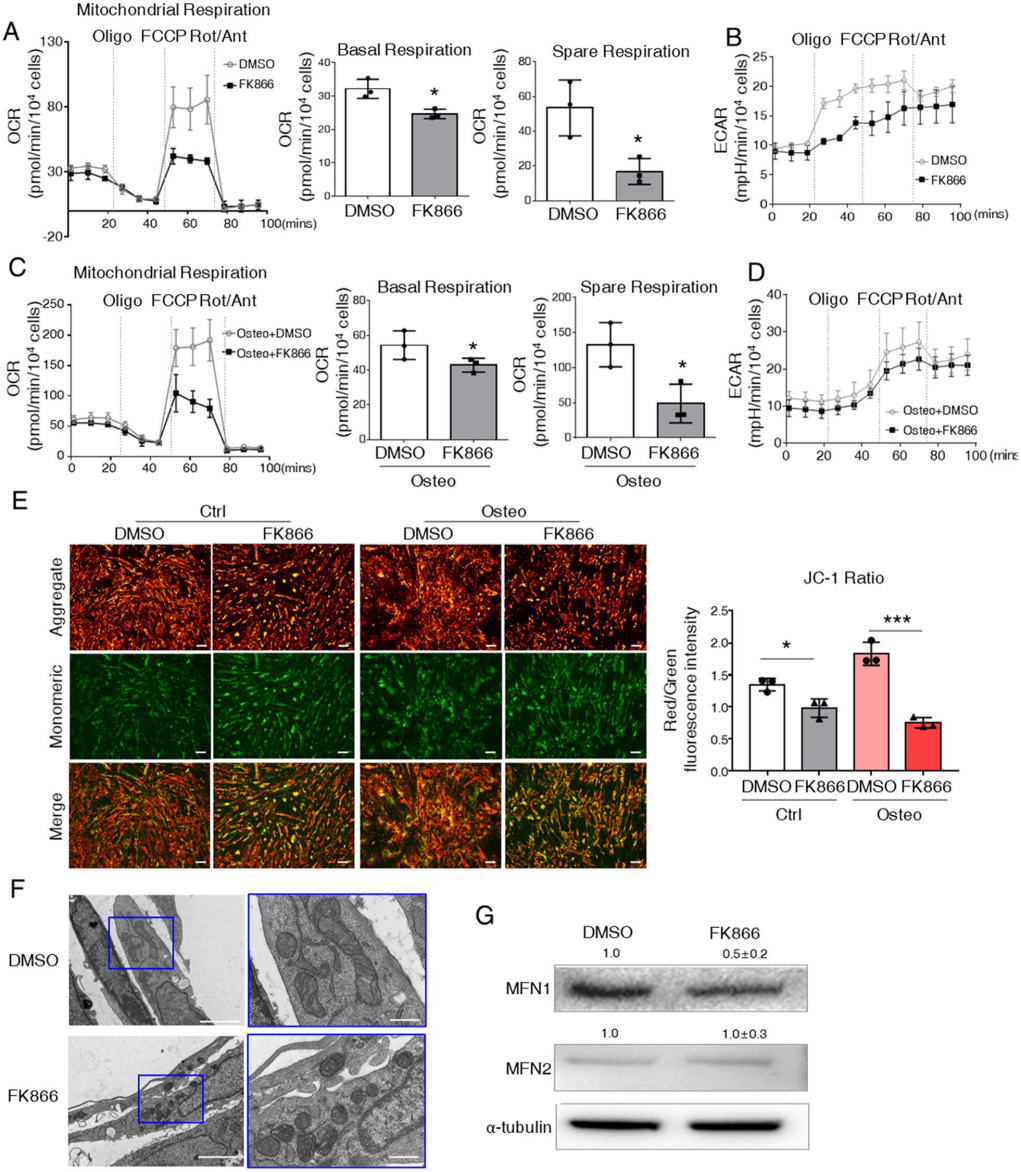
FK866 suppresses mitochondria fusion and OXPHOS. **A**, **C** Seahorse mito-stress test for OCR in undifferentiated ST2 cells (**A**) or osteogenic committed ST2 cells (**C**) at day 7. The right panels: quantification of OCR parameters. **B**, **D** Seahorse mito-stress test for ECAR in undifferentiated ST2 cells (**B**) or osteogenic committed ST2 cells (**D**) at day 7. **E** Representative images of JC-1 staining in ST2 cells. The right panel: quantification of red/green fluorescence intensity ratio in ST2 cells. Scale bar = 100 μm. **F** Representative transmission electron microscope images of mitochondria in osteogenic committed ST2 cells at day 7. Scale bar in the right panel: 2.0 μm, scale bar in the left panel: 500 nm. **G** Representative image of western blot analysis with the osteogenic committed ST2 cells at day 7. For (**A**, **C**), statistical significance was calculated with Student’s t test. For (**E**), statistical significance was calculated with two-way ANOVA. *p < 0.05; **p < 0.01; ***p < 0.001. Data are represented as mean ± SD

To explore further the role of NAD^+^ in metabolic alterations during osteogenesis, we analyzed the OCR and ECAR with FK866. Mitochondrial stress tests showed that basal respiration and maximal respiration were all suppressed by FK866 in both undifferentiated ST2 cells and osteogenic cells (Fig. 5A, C). Specifically, FK866 dramatically suppressed the spare respiration in either undifferentiated or osteogenic cells, suggesting the inhibition of mitochondrial function. However, ECAR did not have significant changes with FK866 treatment in either type of cells (Fig. 5B, D). As activation of OXPHOS and mitochondria function were correlated with osteogenesis, we next investigated the effect of FK866 on mitochondrial potential and morphology. JC1 staining revealed that FK866 diminished mitochondrial membrane potential (Fig. 5E). More importantly, we found that the mitochondria became punctated with FK866 treatment by scanning electron microscope (Fig. 5F). Accordingly, the expression of MFN1 was repressed by FK866 (Fig. 5G).

**Fig. 6:**
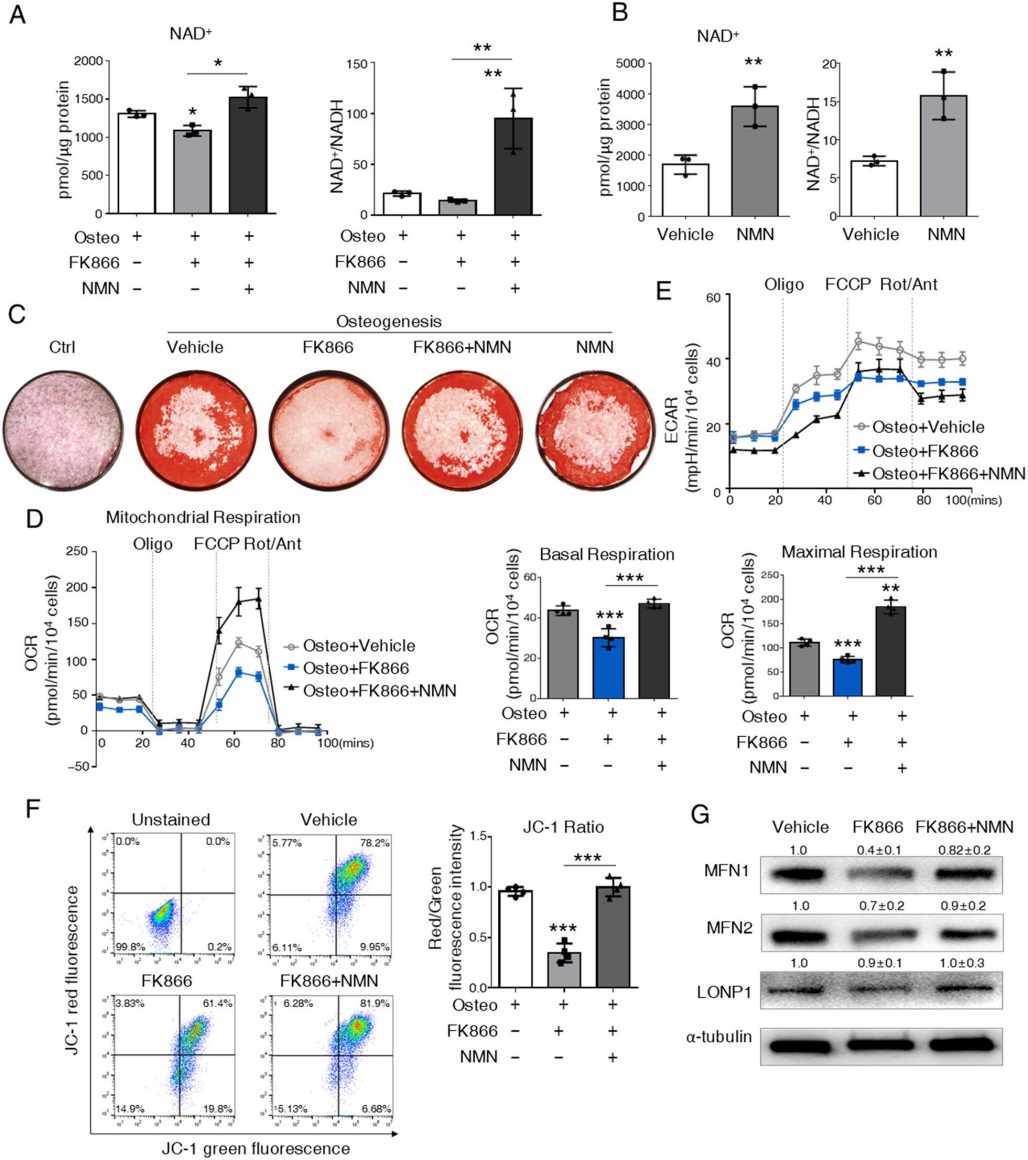
Replenishment of NMN diminishes FK866 inhibition on osteogenic commitment by recovering mitochondrial OXPHOS. **A**, **B** Intracellular level of NAD^+^, and the ratio of NAD^+^/NADH in ST2 cells. **C** Alizarin red stain of ST2 cells at day 7. **D** Seahorse mito-stress test for OCR in osteogenic committed ST2 cells at day 7. The right panels: quantification of OCR parameters. **E** Seahorse mito-stress test for ECAR in osteogenic committed ST2 cells at day 7. **F** FACS analysis and quantification of JC-1 stain in osteogenic committed ST2 cells. The relative ΔΨm measured by the red/green fluorescence intensity ratio are shown. **G** Representative image of western blot analysis with the osteogenic committed ST2 cells at day 7. Quantification of the western blot from three independent experiments was shown upon the bands. *N* = 3. Statistical significance was calculated with one-way ANOVA. *p < 0.05; **p < 0.01, *N* = 3. Data are represented as mean ± SD

NAMPT is known as the rate-limiting enzyme for NAD^+^ salvage synthesis, which catalyzes NAM to NMN. NMN is then converted into NAD^+^ by NMN adenylyltransferases (NMNATs). Since NMN is the immediate product of the NAMPT, we investigated whether NMN could alleviate the inhibition of FK866 on osteogenesis and rescue mitochondria function. As expected, intracellular NAD^+^ level with FK866 treatment could be restored by NMN (Fig. 6A). Supplement of NMN led to a twofold increase in NAD^+^ level (Fig. 6B). Notably, the Alizarin red stain showed that NMN treatment partially recovered osteogenesis and mineralization, which was suppressed by FK866 (Fig. 6C). NMN itself did not promote osteogenesis. Of note, NMN markedly prevented the decrease of basal respiration and maximal respiration by FK866 without any change in ECAR level (Fig. 6D, E). JC1 staining also revealed that NMN improved mitochondrial membrane potential significantly (Fig. 6F). Besides, the expression of MFN1 and MFN2 was elevated by NMN (Fig. 6G). Thus, our data indicated that inhibition of NAD^+^ synthesis by FK866 suppressed mitochondrial OXPHOS mediated-BMSC osteogenesis, which could be partially recovered by the replenishment of NMN.

## Discussion

**Fig. 7:**
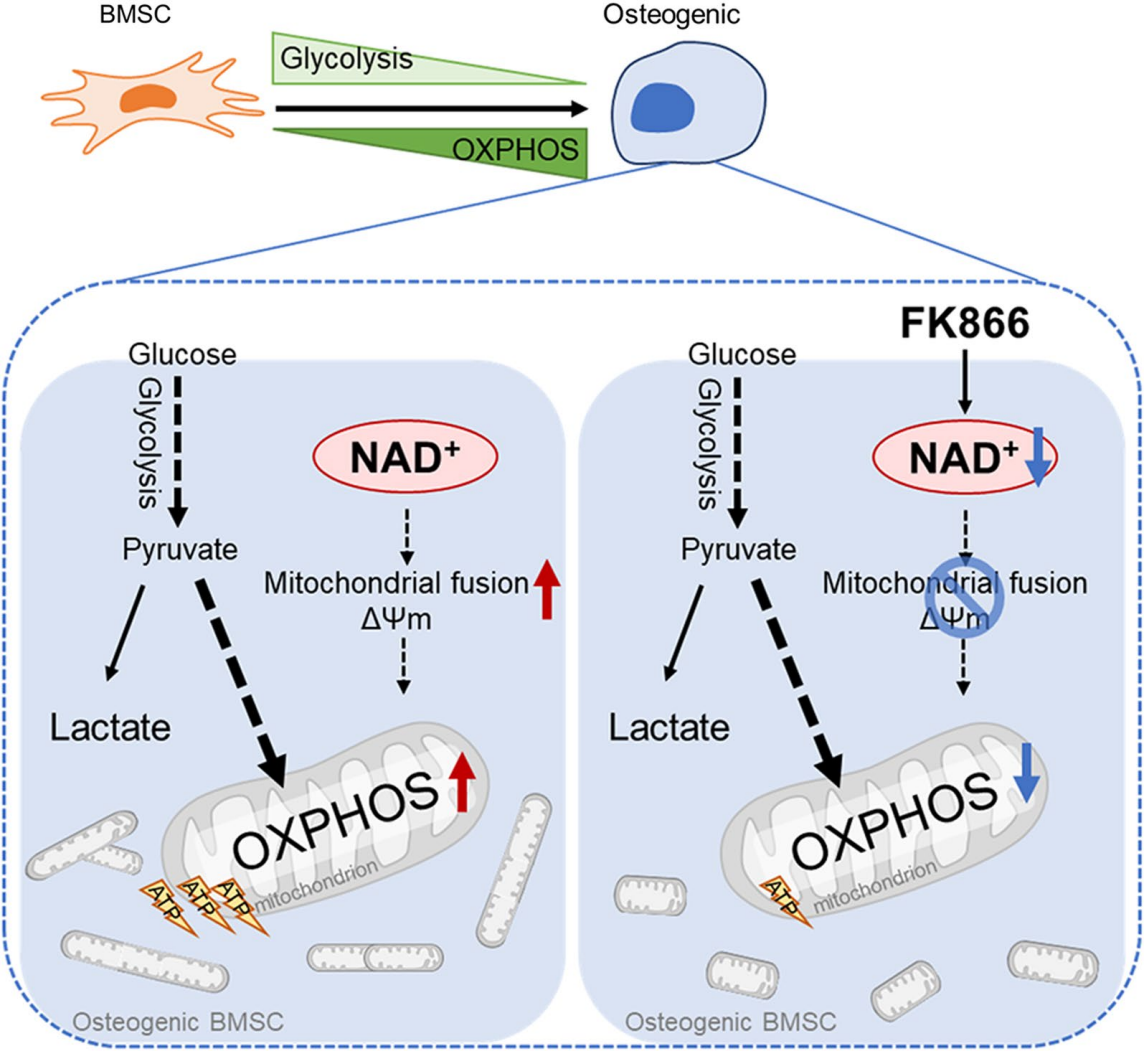
Graphical abstract. Osteogenic committed BMSCs exhibit an elevated intracellular NAD^+^ level associated with an increased oxidative phosphorylation (OXPHOS) activity and a decreased glycolysis. Attenuates of NAD^+^ by FK866 diminish osteogenic commitment of BMSCs and impair bone fracture healing due to mitochondria dysfunction and reduced activity of OXPHOS. Additionally, deficiency of NAD^+^ impairs bone fracture healing

In this work, we aimed to determine the metabolic profile of OXPHOS and glycolysis during BMSCs lineage commitment and the role of NAD^+^ in metabolic regulation. Our data clearly show the evidence that BMSCs undergoing osteogenesis have elevated OXPHOS activity and declined glycolytic activity. Consistent with the enhanced OXPHOS, the morphology of mitochondria becomes slender, the mitochondrial number and membrane potential are dramatically increased during osteogenesis. On the other hand, adipogenic committed BMSCs exhibit increased activity in both OXPHOS and glycolytic. As a vital metabolite, maintaining intracellular NAD^+^ levels is necessary and sufficient for BMSCs osteogenesis. Reducing NAD^+^ level by repressing NAD^+^ synthesis with NAMPT inhibitor FK866 impaired osteogenesis and bone fracture repair, while elevation NAD^+^ level by NAMPT activator P7C3 stimulated osteogenesis. Moreover, our data indicate that the NAD^+^ is necessary to sustain the mitochondrial function and OXPHOS activity (Fig. 7).

In recent years, an increasing number of studies have found that stem cell differentiation is related to energy metabolism. It was reported that glycolysis was preferred in stem cells for energy supply. When cells commit to differentiation, OXPHOS becomes active to meet higher energy demand for differentiation [31]. Meanwhile, the metabolic state may also change upon the different extracellular environments, such as nutrient supplementation. High glucose is a powerful driver of the crabtree effect, which is the obstruction of OXPHOS [32] and the induction of glycolytic flux [33]. Excess pyruvate could accelerate glycolysis [34]. Previous work proves that BMSC-like C3H10T1/2 cells cultured in 5 mM glucose increase mitochondrial OXPHOS while there is no change in glycolytic lactate with osteogenic induction [35]. A recent study also demonstrates the enhanced OXPHOS in stromal cell line ST2 and osteoblast-like MC3T3-E1 cell line with little effect on glycolysis when cells are cultured in osteogenic induction media containing 5.5 mM glucose [36]. Wei et al. reports that the expression of proteins involved in mitochondrial function is increased upon osteogenic induction of MSCs, including PGC-1α, enzymes of the TCA cycle, and protein subunits of respiratory enzymes [37]. Conversely, another study shows that osteoblast-like MC3T3-E1 cells prefer glycolysis rather than OXPHOS to meet ATP demand during osteogenesis, using medium containing 25 mM glucose, 10 mM pyruvate, and 2 mM glutamine [38]. Even cultured with the 5.5 mM glucose, Revollo et al. shows different results that osteogenic induction in hBMSCs triggers a decrease in lactate release without alteration in oxygen consumption (34).

Furthermore, the metabolic profile may vary in different cell types or different stages of differentiation. For instance, undifferentiated human mesenchymal stem cells (hMSCs) show higher levels of glycolytic enzymes and lactate production rate [9]. One study on mouse skin mesenchymal stem cells (msMSCs) reports that adipogenesis and osteogenesis involve enhanced mitochondrial respiration during the early commitment phase [39], whereas the mature calvarial osteoblast and MC3T3-E1 cell line, which represent terminal differentiation, prefer glycolysis for ATP production [12].

In this study, we focused on the mesenchymal stem cell or stromal cell and conducted the glucose, lactate measurements, and seahorse assay within the αMEM containing 5.5 mM glucose, 2 mM glutamine, and 0.1 mM pyruvate to maintain cells in a physiologic state. Besides, following the osteogenic or adipogenic induction for 7 days, the cells were then trypsinized and plated into the XF24 plates 2 h before the seahorse assay to ensure that the cells were in good differentiation condition. Our data support that OXPHOS activity is increased during either osteogenesis or adipogenesis in BMSCs. Here we confirm that the OXPHOS is enhanced in osteogenic and adipogenic differentiation, while the glycolytic activity is declined during osteogenesis but elevated during adipogenesis.

In mammals, NAD^+^ is predominantly synthesized from nicotinamide through the salvage pathway or simply synthesized from tryptophan or aspartic acid, the de novo pathway [40]. Nicotinamide is converted to NMN by NAMPT, the rate-limiting enzyme in the salvage pathway. Our data shows that the NAD^+^ level is increased in osteogenic committed cells and decreased in adipogenic cells, which is consistent with the expression of NAMPT. As a cofactor of sirtuins and Poly (ADP-ribose) polymerase (PARPs), the role of NAD^+^ is mainly concerned with posttranslational modifications in bone homeostasis [20, 21, 41], little is known about the effect of NAD^+^ on cellular metabolism during BMSCs differentiation. NAD^+^ and the redox state of NAD^+^/NADH contribute to metabolic programming. NAD^+^ is essential for mitochondrial ATP production as a coenzyme and affects mitochondrial function [42]. Our calculation of the Seahorse data shows that inhibition of NAD^+^ suppresses the mitochondrial OXPHOS activity. The mitochondrial membrane potential decreases to approximately 33% upon FK866 treatment. And the inhibition of FK866 on mitochondrial function can be rescued by NMN. Besides, the expression of mitochondrial fusion-related genes is repressed with FK866. Interestingly, the literature reports that the regeneration of NAD^+^ from NADH is required to support glycolysis [43]. NAMPT inhibitor FK866 leads to attenuation of glycolysis by blocking the glyceraldehyde 3-phosphate dehydrogenase step [44]. However, our data shows that the basal ECAR level is not altered with FK866 treatment, which may attribute to the remaining portion of NAD^+^. Collectively, we demonstrate that suppression of NAD^+^ impairs BMSCs osteogenesis via manipulating mitochondrial OXPHOS activity.

Bone fracture healing is a multistage process involving various cell lineages, including immune cells, skeletal stem cells, progenitors, and endothelial cells [45]. BMSCs are essential to fracture healing as they can ultimately differentiate into chondrocytes and osteoblasts to regenerate the fractured bone. However, the overall risk of delayed union or non-union is 5 to 10%, mainly caused by the impaired osteogenic capacity [46, 47]. In this regard, cell therapy is considered a promising method to correct this imperfect osteogenesis [48]. In recent years, the understanding of the NAD homeostasis in aging and cancer provides us a new insight into the biological function of NAD^+^. For instance, intracellular NAD^+^ depletion with FK866 effectively suppresses cancer cell proliferation and improves cancer survival [49, 50]. This compound has progressed to phase II clinical trials for anticancer chemotherapy [51]. To evaluate the effect of FK866 on osteogenesis in vivo, we conduct the bone fracture model and intraperitoneal injection with FK866. Consistent with the in vitro data, we show that fracture healing is repressed with the impaired formation of cartilage and bone. Besides, the mineral density of the callus is reduced with the FK866 treatment. It will be of interest to examine whether replenishment of NAD^+^ or the intermediates benefits fracture repair.

## Conclusion

Overall, this study delineated the distinct metabolic profile during the osteogenic and adipogenic commitment of BMSCs. We identified NAD^+^ regulating BMSCs osteogenesis via OXPHOS. The attenuates of NAD^+^ with NAMPT inhibitor FK866 suppressed BMSCs osteogenesis and bone fracture repair. Further experiments will be necessary to examine the potential of NAD^+^ and the intermediates as a therapeutic target for bone repair and regeneration.

## Supplementary Information

**Additional file 1.** **Fig. S1.** Osteogenic commitment and adipogenic commitment of hBMSCs. **A** FACS analysis of hBMSCs. The unstained population was shown in blue. **B** Representative images of Oil Red O staining of hBMSCs at day 7. Scale bar: 100 μm. **C** Representative images of Alizarin Red staining of hBMSCs at day 7. Scale bar: 100 μm. **D**, **E** Relative expression of osteogenic-related genes (**D**) or adipogenic-related genes (**E**). Statistical significance was calculated with Student’s t test. *p < 0.05; **p < 0.01, *N* = 3, error bars: std. **Fig. S2.** The metabolic changes in ST2 cells. **A** Seahorse mito-stress test for OCR, ECAR, and the energy map of ST2 cells on Day 7. **B** Glucose consumption rate in ST2 cells on Day 7. **C** Lactate production rate inST2 cells on Day 7. Ctrl: control; Osteo: osteogenesis; Adipo: adipogenesis; Oligo: oligomycin; FCCP: Trifluoromethoxy carbonylcyanide phenylhydrazone; Rot: rotenone; Ant: antimycin A. *N* = 3. Statistical significance was calculated with one-way ANOVA. *p < 0.05; **p < 0.01. Data are represented as mean ± SD. **Fig. S3.** ALP staining of undifferentiated hBMSCs with FK866 or P7C3 treatment for 7 days. Representative images of the ALP staining of hBMSCs cultured without osteogenic-induction medium for 7 days. The right panel was the quantification of ALP staining density. Statistical significance was calculated with one-way ANOVA. *p < 0.05; **p < 0.01. *N* = 4. Data are represented as mean ± SD. **Fig. S4.** NAMPT expression in bone callus during bone fracture repair. Representative images of the H&E stain and NAMPT expression in bony callus. The yellow dashed line indicates the bone callus. Lower panels show the area in the black box with high magnification. Black arrows indicated the hard callus. Scale bar in low magnification: 200 μm, scale bar in high magnification: 50 μm. *N* = 5

## Data Availability

All data generated or analyzed during the study are included in the article.

## Abbreviations

BMSCs: Bone marrow mesenchymal stem cells
OXPHOS: Oxidative phosphorylation
ATP: Adenosine triphosphate
TCA: Tricarboxylic acid cycle
NAD: Nicotinamide adenine dinucleotide
FAD: Flavin adenine dinucleotide
NAM: Nicotinamide
NMN: Nicotinamide mononucleotide
OCR: Oxygen consumption rate
ECAR: Extracellular acidification rate
GCR: Glucose consumption rate
LPR: Lactate production rate
ΔΨm: Mitochondrial membrane potential
MFN1: Mitofusin-1
MFN2: Mitofusin-2
FIS1: Fission 1
NAMPT: Nicotinamide phosphoribosyltransferase
NMNATs: NMN adenylyltransferases
msMSCs: Mouse skin mesenchymal stem cells
PARPs: Poly (ADP-ribose) polymerase
ALP: Alkaline phosphatase
IBMX: 3-Isobutyl-1-methylxanthine
FCCP: Trifluoromethoxy carbonylcyanide phenylhydrazone
2DG: 2-Deoxy-d-glucose
CCCP: Carbonyl cyanide 3-chlorophenylhydrazone
CS: Citrate synthase
IP: Intraperitoneal
TV: Total volume
BV: Bone volume
BMD: Bone mineral density
