# Supplementary material for: Attenuates of NAD+ impair BMSC osteogenesis and fracture repair through OXPHOS
Source: Stem Cell Res Ther. 2022 Feb 22;13:77. doi: 10.1186/s13287-022-02748-9 (PMC8864833; doi:10.1186/s13287-022-02748-9)
Supplement: Supplementary file 1 — Additional file 1. Fig. S1. Osteogenic commitment and adipogenic commitment of hBMSCs. A FACS analysis of hBMSCs. The unstained population was shown in blue. B Representative images of Oil Red O staining of hBMSCs at day 7. Scale bar: 100 μm. C Representative images of Alizarin Red staining of hBMSCs at day 7. Scale bar: 100 μm. D, E Relative expression of osteogenic-related genes (D) or adipogenic-related genes (E). Statistical significance was calculated with Student’s t test. *p < 0.05; **p < 0.01, N = 3, error bars: std. Fig. S2. The metabolic changes in ST2 cells. A Seahorse mito-stress test for OCR, ECAR, and the energy map of ST2 cells on Day 7. B Glucose consumption rate in ST2 cells on Day 7. C Lactate production rate inST2 cells on Day 7. Ctrl: control; Osteo: osteogenesis; Adipo: adipogenesis; Oligo: oligomycin; FCCP: Trifluoromethoxy carbonylcyanide phenylhydrazone; Rot: rotenone; Ant: antimycin A. N = 3. Statistical significance was calculated with one-way ANOVA. *p < 0.05; **p < 0.01. Data are represented as mean ± SD. Fig. S3. ALP staining of undifferentiated hBMSCs with FK866 or P7C3 treatment for 7 days. Representative images of the ALP staining of hBMSCs cultured without osteogenic-induction medium for 7 days. The right panel was the quantification of ALP staining density. Statistical significance was calculated with one-way ANOVA. *p < 0.05; **p < 0.01. N = 4. Data are represented as mean ± SD. Fig. S4. NAMPT expression in bone callus during bone fracture repair. Representative images of the H&E stain and NAMPT expression in bony callus. The yellow dashed line indicates the bone callus. Lower panels show the area in the black box with high magnification. Black arrows indicated the hard callus. Scale bar in low magnification: 200 μm, scale bar in high magnification: 50 μm. N = 5 [file 13287_2022_2748_MOESM1_ESM.pdf]

Attenuates of NAD^+ impair BMSC osteogenesis and fracture repair through OXPHOS

Boer Li[1,2,3], Yu Shi[1,3], Mengyu Liu[1,2], Fanzi Wu[1], Xuchen Hu[1,2], Fanyuan Yu[1,2], Chenglin Wang[1,2], Ling Ye[1,2,*]

[1]State Key Laboratory of Oral Diseases & National Clinical Research Center for Oral Diseases, West China Hospital of Stomatology, Sichuan University, Chengdu, China.

[2]Department of Endodontics, West China Hospital of Stomatology, Sichuan University, Chengdu, CHINA

[3] These authors contributed equally

[*]Correspondence: yeling@scu.edu.cn

Correspondence

Ling Ye

DDS, Ph.D., Department of Endodontics, West China Stomatology Hospital, Sichuan University, No.14, Section 3, South Renmin Road, Chengdu, China, 610041.

Telephone: 862885503497

Fax: 862885582167

E-mail: yeling@scu.edu.cn

Additional file 1

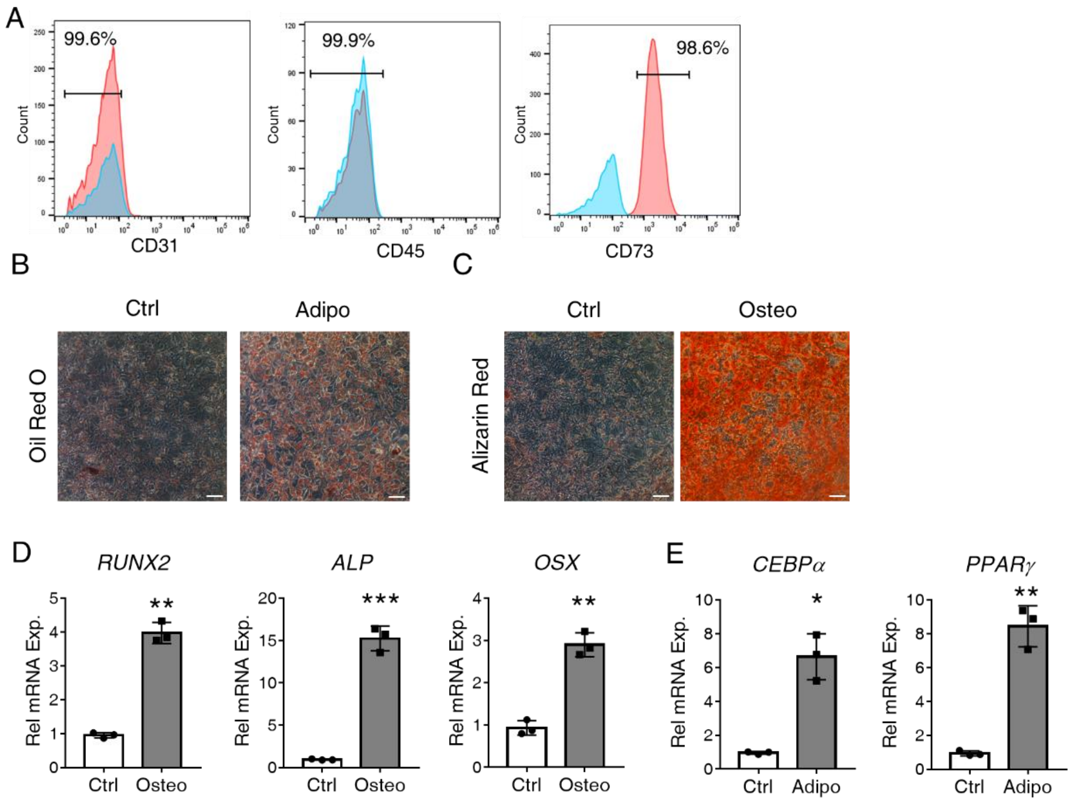

Figure S1. Osteogenic commitment and adipogenic commitment of hBMSCs.

(A) FACS analysis of hBMSC. The unstained population was shown in blue. (B) Representative images of Oil Red O staining of hBMSCs at day 7. Scale bar: 100μm. (C) Representative images of Alizarin Red staining of hBMSCs at day 7. Scale bar: 100μm. (D, E) Relative expression of osteogenic-related genes (D) or adipogenic-related genes (E). Statistical significance was calculated with Student's t test. *: $p<0.05$; **: $p< 0.01$, N=3, error bars: std.

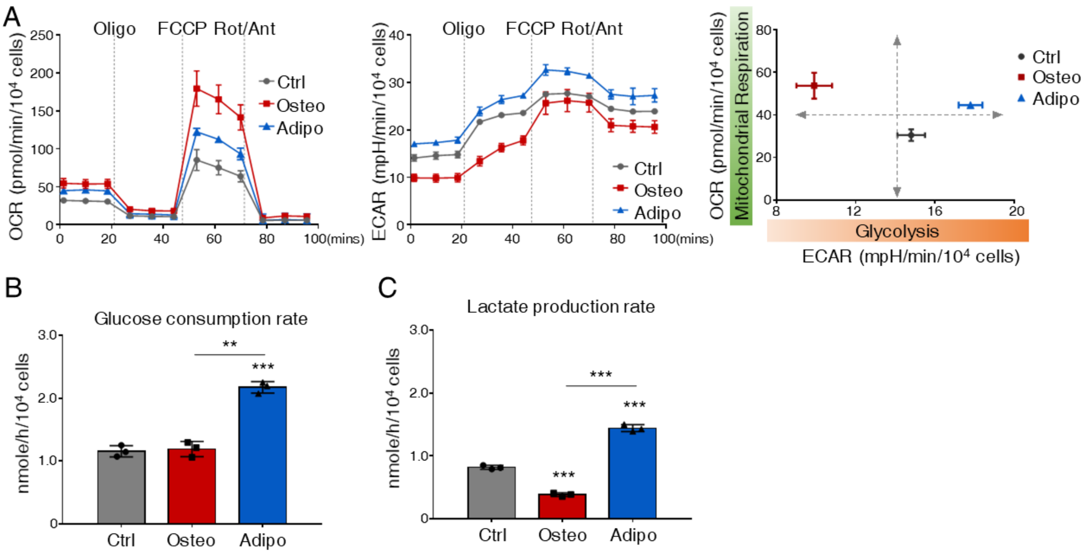

Figure S2. The metabolic changes in ST2 cells.

(A) Seahorse mito-stress test for OCR, ECAR, and the energy map of ST2 cells on Day7. (B) Glucose consumption rate in ST2 cells on Day7. (C) Lactate production rate inST2 cells on Day7. Ctrl: control; Osteo: osteogenesis; Adipo: adipogenesis; Oligo: oligomycin; FCCP: Trifluoromethoxy carbonylcyanide phenylhydrazone; Rot: rotenone; Ant: antimycin A. N=3. Statistical significance was calculated with one-way ANOVA. *: p<0.05; **: p< 0.01. Data are represented as mean ± SD.

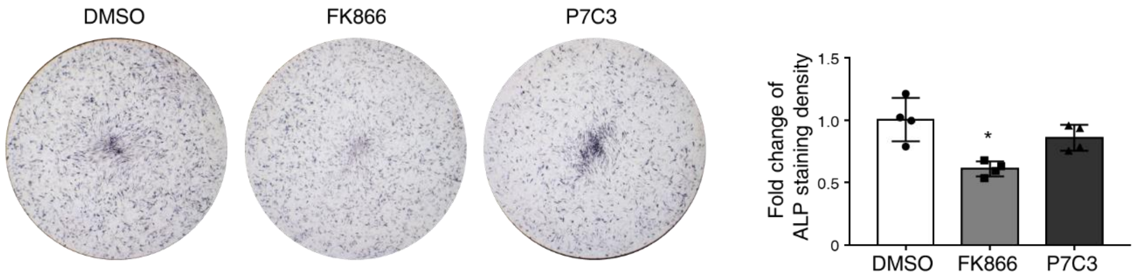

Figure S3. ALP staining of undifferentiated hBMSC with FK866 or P7C3 treatment for 7days.

Representative images of the ALP staining of hBMSC cultured without osteogenic-induction medium for 7 Days. The right panel was the quantification of ALP staining density. Statistical significance was calculated with one-way ANOVA. *: $p<0.05$; **: $p< 0.01$. N=4. Data are represented as mean ± SD.

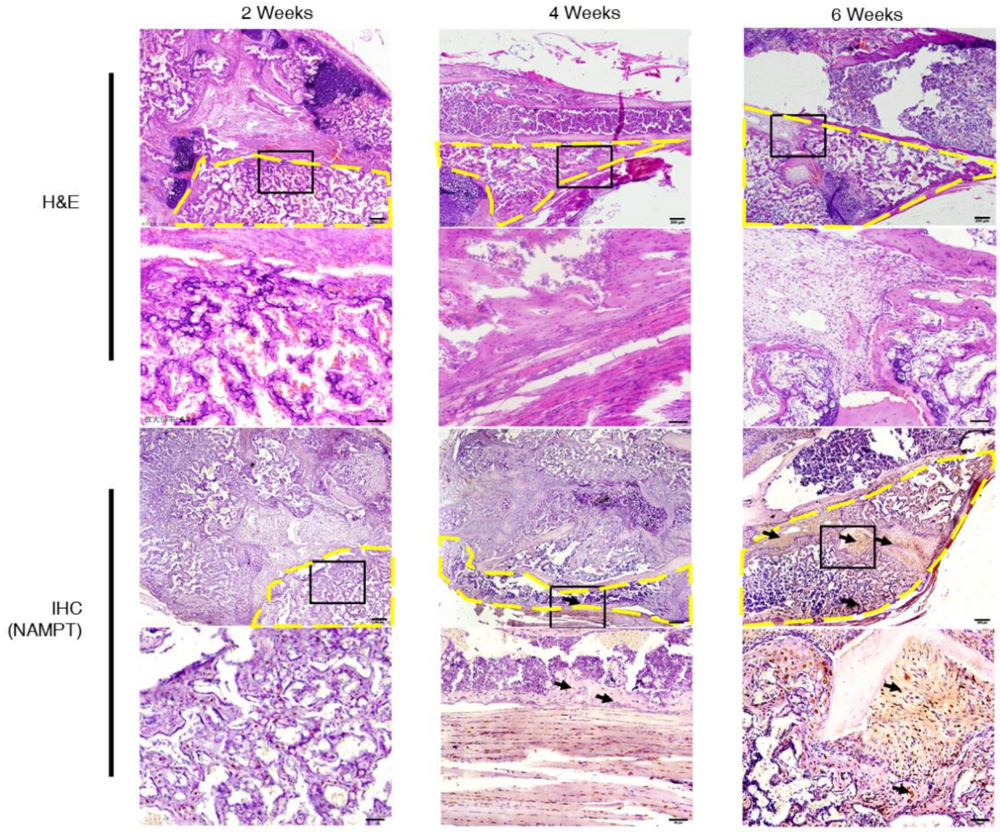

Figure S4. NAMPT expression in bone callus during bone fracture repair. Representative images of the H&E stain and NAMPT expression in bony callus. The yellow dashed line indicates the bone callus. Lower panels show the area in the black box with high magnification. Black arrows indicated the hard callus. Scale bar in low magnification: 200µm, scale bar in high magnification: 50µm. N=5.
